# Inattention and hyperactivity symptoms in childhood predict physical activity in adolescence

**DOI:** 10.1186/s12888-021-03603-6

**Published:** 2021-12-18

**Authors:** Eva Norén Selinus, Natalie Durbeej, Yiqiang Zhan, Paul Lichtenstein, Sebastian Lundström, Maria Ekblom

**Affiliations:** 1 Region Vastmanland - Uppsala University, Centre for Clinical Research, Vastmanland Hospital Vasteras, Uppsala, Sweden; 2 The Swedish School of Sport and Health Sciences, Stockholm, Sweden; 3 Department of Clinical Neuroscience, Centre for Psychiatry Research & Education, Karolinska Institutet & Stockholm County Council, Stockholm, Sweden; 4 Child Health and Parenting (CHAP), Department of Public Health and Caring Sciences, Uppsala University, Uppsala, Sweden; 5 German Center for Neurogenerative Diseases, Ulm, Germany; 6 Institute of Environmental Medicine, Karolinska Institutet, Stockholm, Sweden; 7 Department of Medical Epidemiology and Biostatistics, Karolinska Institutet, Stockholm, Sweden; 8 Center for Ethics, Law and Mental Health (CELAM), University of Gothenburg, Göteborg, Sweden; 9 Gillberg Neuropsychiatry Centre, University of Gothenburg, Göteborg, Sweden; 10 Department of Neuroscience, Karolinska Institutet, Stockholm, Sweden

**Keywords:** ADHD, Hyperactivity, Inattention, Longitudinal, Neurodevelopmental problems, Physical activity

## Abstract

**Background:**

Physical activity has been documented to influence several aspects of physical and mental health. Growing evidence shows that physical activity can improve attention. Less is known about how symptoms of inattention and hyperactivity / impulsivity in childhood are associated with physical activity in adolescence. We aimed to explore this relationship further.

**Methods:**

We used a cohort of 3949 Swedish children (1884 boys and 2065 girls) with data collected at ages 9 (or 12) and 15. We investigated the influence of symptoms of inattention and hyperactivity / impulsivity in childhood – age 9/12 (inattention and hyperactivity/impulsivity separately) on self-rated physical activity at age 15, using multiple logistic regression models. We considered potential confounders such as sex, parental education level, physical activity in childhood and neurodevelopmental comorbidity. A cluster robust sandwich estimator was applied to adjust the standard errors for the nested twin data when computing the regression models.

**Results:**

Symptoms of inattention in childhood (9/12) predicted less physical activity in adolescence (age 15) (OR = 0.83 CI = 0.78–0.89), whereas the opposite was true for hyperactivity/impulsivity (OR = 1.08 CI = 1.02–1.10). These associations still remained when taking possible confounders into account including neurodevelopmental and neurodevelopmental related comorbidity.

**Conclusions:**

These findings support the importance of helping children and adolescents with inattention symptoms to engage in physical activity in suitable settings.

## Introduction

Physical activity is an important life style factor for many health outcomes throughout life. While the most commonly known health effects include cardiovascular health and metabolic health, a number of recent studies have suggested that physical activity might also benefit brain health. WHO recommends that children and adolescents should do at least an average of 60 min per day of moderate-to vigorous-intensity, mostly aerobic, physical activity, across the week, and that vigorous-intensity aerobic activities, as well as those that strengthen muscle and bone should be incorporated at least 3 days per week [1].

In cross-sectional studies, physical activity and aerobic fitness have been shown to be positively associated to children’s cognitive function and academic achievements [2, 3]. In Sweden schools, parents and other parts of society have showed a growing interest in long-term health consequences of symptoms of inattention and hyperactivity / impulsivity. The prevalence of ADHD among Swedish school-aged children has been estimated by the National Board of Health and Welfare to be around 3 to 5%, based on a review of Swedish and international studies in 2002 [4]. However, since then another study has concluded that increasing numbers of children in Sweden are being diagnosed with ADHD and that clinically diagnosed ADHD increased more than fivefold from 2004 to 2014 [5]. Furthermore, other coexisting neurodevelopmental problems are also quite common among children with ADHD [6].

Children with ADHD in ages 6–17 years tend to be less involved in physical activity and organized sports than their peers [7], and are almost twice as likely to have fewer healthy behaviors than their peers [8].

While in cross sectional data, a cause and effect relationship between ADHD and associated factors cannot be deduced, longitudinal studies can be used to study how symptoms and lifestyles develop over time.

In an attempt to study the causality between physical activity and symptoms of inattention and hyperactivity / impulsivity, Rommel et al. used twin-analysis in a longitudinal study between adolescence and young adulthood. They found that physical activity in adolescence was inversely associated with ADHD symptoms in young adulthood, even after adjusting for unmeasured confounding, suggesting that physical activity might indeed mitigate future symptoms of inattention and hyperactivity / impulsivity [9]. When studying a younger age group, less sedentary behavior at age 7 has on the other hand been shown to predict ADHD diagnosis at age 14 [10]. The findings that more activity in adolescence is associated with fewer symptoms of ADHD in adulthood does not necessarily contradict the finding that less sedentary behaviour in childhood predicts higher ADHD symptoms in adolescence. These studies involve two different age groups, which can explain differing results over time.

Since ADHD is highly heritable [11], it is not uncommon that a child with ADHD has a parent who also has ADHD. It has been shown that adult ADHD is associated with functional impairment with consequences such as lower educational attainment and lower level of employment [12]. This may influence the parent’s capacity to support a child’s daily life in an optimal way to create healthy behaviours and habits.

Several studies have shown associations between ADHD and poor health outcomes [13, 14], but less is known about how the specific symptoms of inattention, hyperactivity, and impulsivity might differ in influencing health enhancing behaviors such as physical activity.

Khalife et al., provided evidence that teacher-reports of ADHD symptoms or conduct disorder symptoms in 8 year old children were associated with an increased risk for being less physically active in adolescence [15]. The findings of Khalife et al., provides initial support for the notion that children with such symptoms might be of increased risk of being less physically active, but such findings could be specific to the Finnish society, and hence need to be confirmed using validated questionnaires in more populations. In Sweden, the government supports the Swedish Sports federation so that they will be able to provide young individuals with good and equal opportunities to participate in sports. A better understanding of how childhood symptoms of inattention and hyperactivity may predict physical activity in adolescence will be important information for health care, schools, parents and sport organizations aiming to provide children with equal opportunities to health promoting lifestyles.

## Aims of the study

We aimed to explore how symptoms of inattention and hyperactivity/impulsivity in childhood are associated with self-rated physical activity in adolescence in a nationally representative sample of Swedish adolescent twins, while controlling for sex, age, parental education, and comorbidity with other neurodevelopmental problems (NDPs). Based on the findings of Khalife et al., we expected to find that more symptoms of inattention and hyperactivity / impulsivity in childhood would be positively associated with self-rating of less physical activity in adolescence [15].

## Material and methods

### Participants

The Child and Adolescent Twin Study in Sweden (CATSS) is an ongoing longitudinal cohort study that follows all twins born in Sweden from 1992 and onwards [16]. It started in the year 2004, and all parents to twins in Sweden are invited to participate in the study through a telephone interview. Before July 2005, parents who consented were interviewed when the twins were 12 years old, but from July 2005 and onwards parents are interviewed when their twins are 9 years old. The CATSS interview covers a broad range of symptoms and functions. When the twins are 15 years old, all families are once again invited to participate in a follow-up survey to fill out written questionnaires (both twins and parents: CATSS-15).

The current study sample was derived from a cohort including 4635 twins who were born 1993–1997, and where data were collected from both data collection waves (parent telephone interview at age 9/12 and questionnaires at age 15). Among the 4635 twins, we included those with complete data on certain variables of interest (see below), i.e., exposures, covariates and outcomes. There were 1968 twin-pairs included in the study. In some other pairs only one of the twins had full data (*n* = 13). A total of 686 twins (15%) had incomplete data on any of the variables and were therefore excluded from the current study. The final study sample thus included 3949 twins (1884 boys and 2065 girls).

The total response rate in this study for both child and parent (i.e. twins where both parent and child had responded in the two data collection waves) at age 15 was 44.1%.

### Measures

#### ADHD symptoms (at age 9/12)

The children included did not receive a clinical diagnosis of ADHD. ADHD was assessed as symptoms of inattention and hyperactivity/impulsivity in a manner commonly employed in population studies.

##### Parent-assessment

Attention deficit hyperactivity disorder symptoms at baseline were assessed using the Autism-Tics, ADHD, and other Comorbidities Inventory (A-TAC) - a diagnostic telephone interview validated for the Swedish population, with modules addressing DSM-IV [17] criteria, and with supplementary symptoms characteristic for the disorders in addition. In previous validation studies, A-TAC has proven to have good-excellent psychometric properties [18], and also convergent validity with the Child Behaviour Check List [19]. ADHD symptoms response categories were scored as 0 (no), 0.5 (to some extent), and 1 (yes). For the dichotomized variable of the total symptoms of ADHD in A-TAC, a cutoff at 6 was used. This has earlier been found to be appropriate in population studies [20].

The inattention scale (0 to 9 points) and hyperactivity/impulsivity scale (0 to 9 points) were used separately as continuous variables.

#### Other NDPs (at age 9/12)

In this study, other NDPs refer to autism spectrum disorder (ASD), learning disorder (LD), tics disorder (TD), and developmental coordination disorder (DCD). Beside these, NDP related problems were also accounted for, such as conduct disorder (CD), oppositional defiant disorder (ODD), obsessive compulsive disorder (OCD), and eating disorder (ED). In the A-TAC interview, cut-off scores for symptoms of the different NDPs were determined and validated in two earlier studies in the Swedish population [21, 22]. Besides for ADHD (as mentioned above), a dichotomous variable was created for each NDP. The ASD cut-off level was set at ≥4.5 (out of 17 points), LD at ≥3.5 (out of 9), TD at ≥2 (out of 4), DCD at ≥1.5 (out of 5). Also, for NDP related problems: CD cut-off level was set at ≥1, ODD at ≥2, OCD at ≥1, and ED at ≥1.5 [22].

#### Physical activity (at age 9)

##### Parent-assessment

“How much time in total does your child need to walk, cycle and/or run to and from school *(per day)*? “1) Less than 15 min; 2) 15–30 min; 3) Half an hour to 1 h; 4) More than an hour. We used the Median, which was 2, as the cut-off to dichotomize the variable ‘Time spent to walk, cycle and/or run to and from school’ (i.e. ≥ 15 min - more than 1 h).

“How often does your child exercise in his/her spare time?” 1) Never; 2) Less than once a month; 3) 1–2 times/month; 4) 1 time/week; 5) 2–3 times/week; 6) 4–5 times/week; 7) Almost every day. We used the Median, which was 5, as the cut-off to dichotomize the variable ‘Occurrence of exercise/work out on spare time’ (i.e. ≥ 2–3 times/week – almost every day).

#### Physical activity (at age 15)

##### Self-assessment

“Which of the following people are you most alike?” 1) moves just a little; 2) moves a lot, but never gets out of breath or sweaty; 3) moves a lot, sometimes gets out of breath and sweaty; 4) moves, gets out of breath and sweaty several times/week; 5) moves, gets out of breath and sweaty every day. We used the Median, which was 3, as the cut-off, in order to define ‘Physical activity’. Rating 4 or higher was defined as being physically active, since this was the alternative appearing to be most in line with the recently updated WHO global recommendations for physical activity for children aged 5–17 [1].

#### Parental education (at age 15)

Data on the parents’ educational level was collected from the parent-reports at age 15 and described in four levels: 1) Compulsory school (9 years)/ Dropped out of compulsory school (< 9 years); 2) 2–4 years of upper secondary school; 3) University degree; 4) Other education. This data was recorded for both parents separately.

### Analyses

Descriptive statistics including means (M), medians (Md), standard deviation (SD), interquartile ranges (IQR), proportions and frequencies were used to describe the sample. We investigated the influence of ADHD symptoms in childhood – age 9/12, focusing on inattention and hyperactivity/impulsivity symptoms, on self-rated physical activity at age 15, using multiple logistic regression models. We considered potential confounders such as physical activity at age 9/12, NDPs and NDP related problems (as mentioned above), sex, age, and parental education level. The variables were entered in three different models with 1) inattention and hyperactivity/impulsivity symptoms, 2) symptoms and confounders including sex, age, parental education, and physical activity at age 9/12, and 3) symptoms and NDPs and NDP related problems added to the other confounders. The estimated associations are reported as odds ratios with 95% confidence intervals (CI). A cluster robust sandwich estimator was applied to adjust the standard errors for the nested twin data when computing the regression models.

Prior to computing the logistic regressions, data were checked for multicollinearity through examining variance inflation factor (VIF) values for all independent variables. VIF-values ≥10 indicates multicollinearity [23]. The VIF-values for the independent variables in the study ranged from 1.03 to 1.73. Thus, multicollinearity was not present in the data. In addition, we checked the assumption of linearity between the logit of the outcome and the inattention and hyperactivity/impulsivity symptoms scales using the Box–Tidwell approach (i.e., an interaction term between each scale and its natural logarithm was added to the logistic regression model) [24]. The associations between the interaction terms and the outcome were nonsignificant (*p* = 0.08 and *p* = 0.67). Thus, none of the inattention or hyperactivity/impulsivity symptoms scales violated the linearity assumption.

All *p*-values equal to or less than 0.05 were considered statistically significant. The software packages SPSS version 23, and STATA version 14 were used in all analyses.

## Results

### Descriptive statistics

We compared the twins included (*n* = 3949) with those excluded (*n* = 686) regarding sex and parental level of education. A larger proportion of the twins included were girls (52.3%) compared to those excluded (46.4%) (X^2^(14635) = 8.24, *p* < .05). However, there were no significant differences between the groups regarding father’s level of education ((X^2^(34635) = 10.36, *p* = .07) or mother’s level of education ((X^2^(34635) = 23.20, *p* = .06). In the total sample, 246 children (6.2%) were screen-positive for ADHD symptoms at age 9/12. Furthermore, a total of 2624 children (66.4%) were physically active at age 15 (self-report). As shown in Table 1, with data referring to the ages of 9/12, the mean scale scores of the Inattention and Hyperactivity/Impulsivity scales were 0.77 and 0.61 points, respectively in the total sample. In addition, the median scores for both scales were 0.00. For about half, the maternal education level was a university degree, and the paternal education level was upper secondary school. About 8 % were screen positive for a learning disorder, and about 1 % were screen positive for OCD and ODD.

**Table 1:** Sample descriptives at age 9/12 in the total sample (*n* = 3949), children physically active at age 15 (self-report) (*n* = 2624) and children not physically active at age 15 (self-report) (*n* = 1325)

	Total sample (n = 3949)	Children physically active at age 15 (n = 2624)	Children not physically active at age 15 (n = 1325)
**Variables**			
**ADHD symptoms**	**M (SD), Md (IQR)**	**M (SD), Md (IQR)**	**M (SD), Md (IQR)**
Inattention symptoms	0.77 (1.40), 0.00 (1.00)	1.28 (0.67), 0.00 (1.00)	0.97 (1.60), 0.00 (2.00)
Hyperactivity/Impulsivity symptoms	0.61 (1.21), 0.00 (1.00)	1.19 (0.60), 0.00 (1.00)	0.64 (1.26), 0.00 (1.00)
**Covariates**	**n (%)**	**n (%)**	**n (%)**
Sex			
Boys	1884 (47.7)	1328 (50.6)	556 (42.0)
Girls	2065 (52.3)	1296 (49.4)	769 (58.0)
Age			
9	1492 (37.8)	1617 (61.6)	840 (63.4)
12	2457 (62.2)	1007 (38.4)	485 (36.6)
Mother’s level of education			
University degree	1894 (48.0)	1331 (50.7)	563 (42.5)
Compulsory school/dropped out of compulsory school	157 (4.0)	97 (3.7)	60 (4.5)
Upper secondary school 2–4 years	1618 (41.0)	1030 (39.3)	588 (44.4)
Other education^1^	280 (7.1)	166 (6.3)	114 (8.6)
Father’s level of education			
University degree	1321 (33.5)	922 (35.1)	399 (30.1)
Compulsory school/dropped out of compulsory school	421 (10.7)	229 (8.7)	192 (14.5)
Upper secondary school 2–4 years	1988 (50.3)	1337 (51.0)	651 (49.1)
Other education^1^	219 (5.5)	136 (5.2)	83 (6.3)
Time spent per day to walk, cycle and/or run to and from school, age 9			
Less than 15 min	1648 (41.7)	1085 (41.3)	563 (42.5)
15 min to more than one hour	2301 (58.3)	1539 (58.7)	762 (57.5)
Occurrence of exercise/work out on spare time, age 9			
About one occasion per week or less	470 (11.9)	220 (8.4)	250 (18.9)
Two-three times per week to almost every day	3479 (88.1)	2404 (91.6)	1075 (81.1)
Screen positive for NDP^2^ problems			
Tic disorder	53 (1.3)	28 (1.1)	25 (1.9)
Learning disorder	330 (8.4)	176 (6.7)	157 (11.6)
Autism spectrum disorder	66 (1.7)	29 (1.1)	37 (2.8)
Developmental coordination disorder	38 (1.0)	19 (0.7)	19 (1.4)
Screen positive for NDP-related problems			
Obsessive-compulsive disorder	40 (1.0)	24 (0.9)	16 (1.2)
Oppositional defiance disorder	47 (1.2)	25 (1.0)	22 (1.7)
Eating disorder	10 (0.3)	4 (0.2)	6 (0.5)
Conduct disorder	4 (0.1)	4 (0.2)	0 (0.0)

^1^ E.g, boarding school, professional school or polytechnic education

^2^ NDP = Neurodevelopmental problem

Children who were physically active had slightly higher mean scores on the Inattention and Hyperactivity/Impulsivity scales, than children who were not physically active. Those who were physically active comprised a larger proportion of boys (50.6%), and had mothers (50.7%) and fathers (35.1%) with a university degree, to a larger extent, than children who were not physically active (boys: 42.0%, mothers with a university degree: 42.5%, fathers with a university degree: 30.1%). The latter comprised a larger proportion who were screen positive for a learning disorder (11.6%) than children who were physically active (6.7%).

### The relation between ADHD symptoms and physical activity

**Fig. 1:**
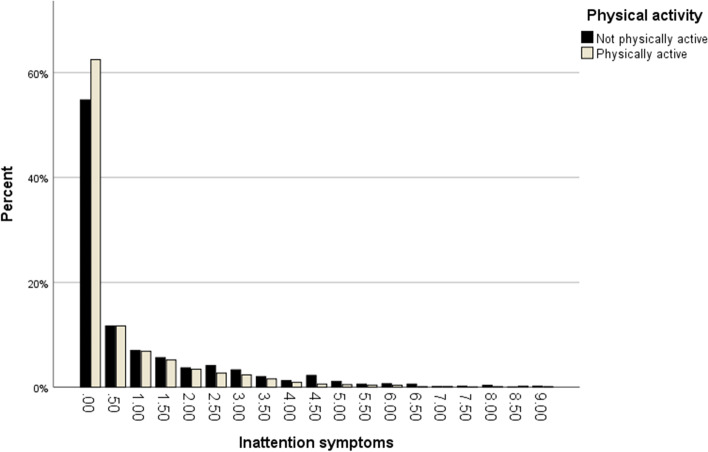
Proportions of participants being physically active and not being physically active at each score level of the Inattention symptoms scale

**Fig. 2:**
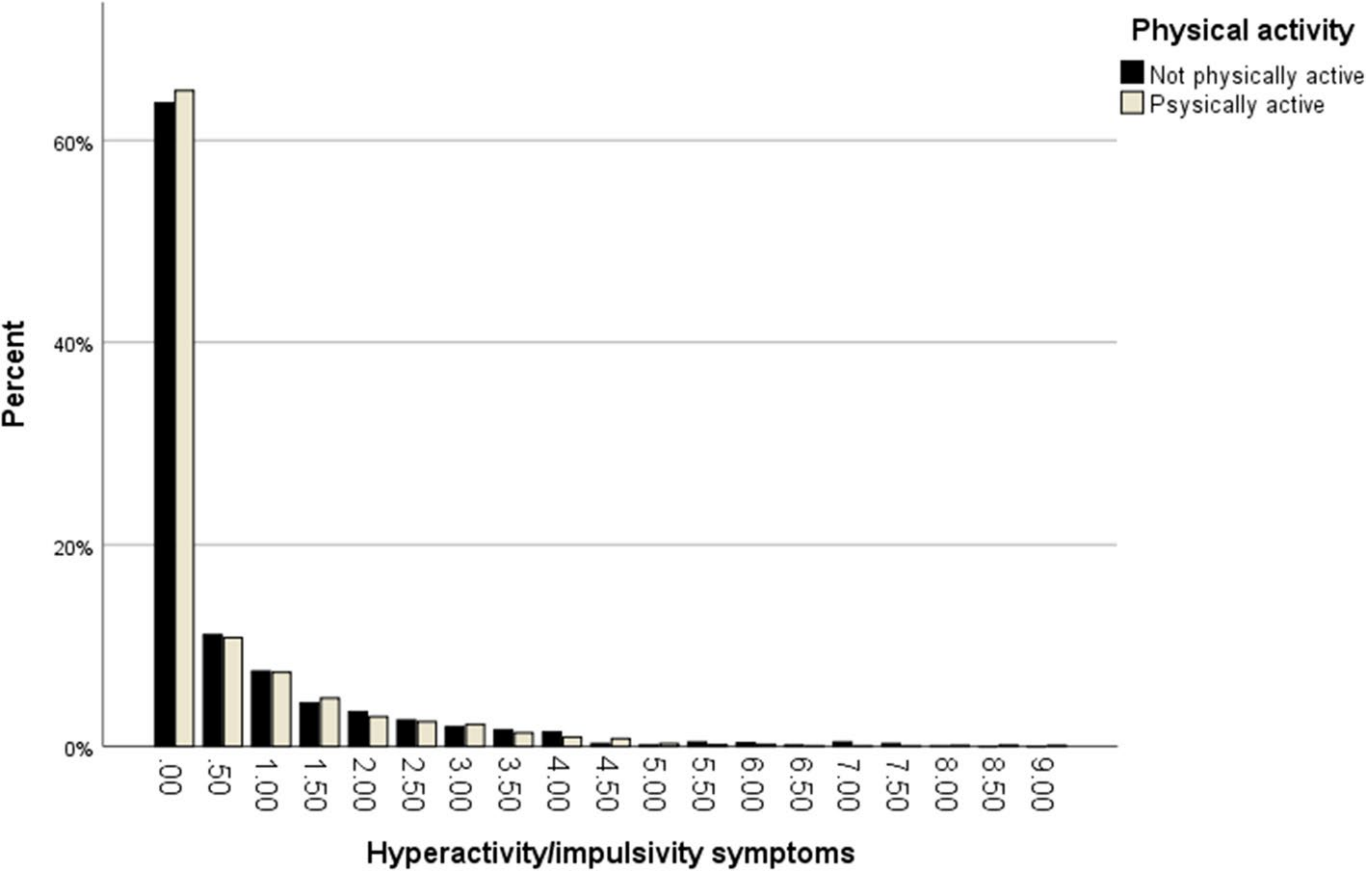
Proportions of participants being physically active and not being physically active at each score level of the Hyperactivity/impulsivity symptoms scale

Bar-charts were drawn in order to visually display the relation between ADHD symptoms and physical activity at age 15. As demonstrated in Figs. 1 and 2, most participants who were physically active scored 0 on both the Inattention (*n* = 1639, 62.5%) and Hyperactivity/impulsivity (*n* = 1704, 64.9%) symptoms scales. This was also the case for participants who were not physically active. (Inattention: *n* = 726, 54.8%; Hyperactivity/impulsivity: *n* = 844, 63.7%).

As shown in Table 2, the unadjusted model demonstrated that higher levels of inattention symptoms in childhood (age 9/12) was associated to a lower likelihood of being physically active in adolescence (age 15), while higher levels of symptoms of hyperactivity/impulsivity in childhood was related to a higher likelihood of being physically active in adolescence.

**Table 2:** Logistic regressions for the associations between Inattention and Hyperactivity/impulsivity (at age 9/12) symptoms and self-rated physical activity (at age 15) (*n* = 3949)

Physical activity (self-rated at age 15)		
Model I^**1**^	***p***	OR (95% Cl)
Inattention symptoms	< .001	0.83 (0.78–0.89)
Hyperactivity/impulsivity symptoms	< .001	1.09 (1.08–1.10)

^1^ The Inattention and Hyperactivity/impulsivity scales were entered simultaneously in this model

When adjusting for sex, physical activity at age 9, and parental education level, these associations remained largely unchanged (Table 3).

**Table 3:** Logistic regressions for the associations between Inattention and Hyperactivity/impulsivity (at age 9/12) symptoms and self-rated physical activity (at age 15), controlling for sex, age, parental education and physical activity at age 9/12 (n = 3949)

Physical activity (self-rated at age 15)		
Model II^**1**^	***p***	OR (95% Cl)
Inattention symptoms	< .001	0.84 (0.79–0.89)
Hyperactivity/impulsivity symptoms	< .001	1.08 (1.07–1.10)
Sex		
Boy (ref)		
Girl	< .001	0.70 (0.68–0.73)
Age		
9 (ref)		
12	< .001	1.09 (1.06–1.12)
Mother’s level of education		
University (ref)		
Compulsory school/dropped out of compulsory school	.201	0.87 (0.70–1.08)
Upper secondary school 2–4 years	.057	0.80 (0.64–1.01)
Other^2^	< .001	0.66 (0.62–0.70)
Father’s level of education		
University (ref)		
Compulsory school/dropped out of compulsory school	< .001	0.63 (0.51–0.78)
Upper secondary school 2–4 years	.831	0.99 (0.89–1.10)
Other^2^	< .001	0.81 (0.78–0.84)
Time spent per day to walk, cycle and/or run to and from school, age 9		
Less than 15 min (ref)		
15 min to more than one hour	.018	1.06 (1.01–1.12)
Occurrence of exercise/work out on spare time, age 9		
About one occasion per week or less (ref)		
Two-three times per week to almost every day	< .001	2.24 (2.03–2.48)

^1^ The Inattention and Hyperactivity/impulsivity scales were entered simultaneously in this model

^2^ E.g, boarding school, professional school or polytechnic education

Furthermore, when adjusting for coexisting NDPs and NDP related problems, the associations also remained (Table 4).

**Table 4:** Logistic regressions for the associations between Inattention and Hyperactivity/impulsivity (at age 9/12) symptoms and self-rated physical activity (at age 15), controlling for sex, age, parental education and physical activity at age 9/12, NDP and non-NDP-related problems at age 9/12 (*n* = 3949)

Physical activity (self-rated at age 15)		
Model III^**1**^	***p***	OR (95% Cl)
Inattention symptoms	< .001	0.87 (0.81–0.93)
Hyperactivity/impulsivity symptoms	< .001	1.10 (1.03–1.18)
Sex		
Boy (ref)		
Girl	< .001	0.70 (0.68–0.72)
Age		
9 (ref)		
12	< .001	1.09 (1.06–1.12)
Mother’s level of education		
University (ref)		
Compulsory school/dropped out of compulsory school	.153	0.87 (0.72–1.05)
Upper secondary school 2–4 years	.086	0.81 (0.64–1.03)
Other^2^	< .001	0.67 (0.63–0.72)
Father’s level of education		
University (ref)		
Compulsory school/dropped out of compulsory school	< .001	0.63 (0.51–0.77)
Upper secondary school 2–4 years	.853	0.99 (0.88–1.11)
Other^2^	< .001	0.82 (0.79–0.85)
Time spent per day to walk, cycle and/or run to and from school, age 9		
Less than 15 min (ref)		
15 min to more than one hour	.037	1.06 (1.00–1.12)
Occurrence of exercise/work out on spare time, age 9		
About one occasion per week or less (ref)		
Two-three times per week to almost every day	< .001	2.22 (2.02–2.45)
Screen positive for NDP problems		
Tic disorder	.064	0.68 (0.46–1.02)
Learning disorder	< .001	0.75 (0.67–0.83)
Autism spectrum disorder	< .001	0.64 (0.50–0.81)
Developmental coordination disorder	.899	0.95 (0.46–1.98)
Screen positive for NDP-related problems		
Obsessive-compulsive disorder	.430	1.11 (0.86–1.43)
Oppositional defiance disorder	.043	0.77 (0.60–0.99)
Eating disorder	.108	0.60 (0.32–1.12)

^1^ The inattention and Hyperactivity/impulsivity scales were entered simultaneously in this model

^2^ E.g, boarding school, professional school or polytechnic education

As expected, in the fully adjusted models (Table 4) girls were less likely to be physically active (OR = 0.70 CI = 0.68–0.72), parental education was of importance, and physical activity during spare time at age 9 was associated with higher odds of being physically active at age 15 (OR = 2.22 CI = 2.02–2.45). Other significant associations are shown in detail in Table 4.

## Discussion

The present study aimed to assess the associations between symptoms of inattention and hyperactivity / impulsivity in childhood and physical activity in adolescence. We explored this relationship in a nationally representative sample of Swedish adolescent twins of both sexes with childhood symptoms of inattention and hyperactivity / impulsivity while controlling for sex, parental education, parent-rated physical activity at age 9/12 and NDP comorbidity.

The main findings were that inattentive symptoms in childhood were associated with being less likely to be physically active in adolescence, and that hyperactivity/impulsivity symptoms in childhood were associated with a higher likelihood of being physically active in adolescence. These associations remained after adjusting for sex, age, parental education level and parent-rated physical activity at age 9/12, and coexisting NDPs and NDP related problems at age 9/12. Nonetheless, our results also indicated a potential floor effect of the symptom scales in relation to physical activity, as most participants who were physically active scored 0 on both scales.

Overall, the expected results were that more symptoms of ADHD in childhood would be positively associated with self-rating of less physical activity in adolescence. Khalife et al. had earlier found such an association [15]. Furthermore, when a child with high levels of symptoms of inattention enters into adolescence, it seems more likely that he/she doesn’t want to adapt, or is incapable of adapting, to regular physical training routines in a group setting or by him−/herself [25]. The impairment that the influence of ADHD symptoms leads to, can also manifest more powerfully during adolescence and result in comorbidity with depression, anxiety, behavioral problems, and bullying. These co-occurring problems would lessen the likelihood of being physically active even more.

The prevalence and social consequences of being diagnosed with ADHD might differ over time and between countries [26]. Recent cross-sectional investigations reported that American children with ADHD were less involved in physical activity than their peers [7], and that children with ADHD were almost twice as likely to have fewer healthy behaviors than peers [8]. However, these studies did not have a longitudinal design.

Our findings of a prospective association between childhood symptoms of inattention and hyperactivity / impulsivity and adolescent physical activity among Swedish twins are supported by results from a longitudinal study by Khalife et al. They found that in a Finnish context, childhood ADHD symptoms and conduct disorder symptoms were linked to being less physically active in adolescence [15]. In these two Nordic contexts therefore, children with inattention symptoms appear to be at increased risk of becoming less physically active in youth. These findings are of great importance for schools, sports clubs, parents and health care workers trying to promote health enhancing physical activity and participation in sports for all.

In the analysis, we controlled for sex. This suggests that despite ADHD being more commonly seen in boys [27, 28], and despite that girls have been shown to be less physically active [29, 30], the association found here between childhood symptoms of inattention and hyperactivity / impulsivity and adolescence physical activity cannot be attributed to sex differences.

As expected, physical activity at age 9/12 in the form of ‘spare time physical activity’ was associated with higher odds of being physically active at age 15. This finding concurs with earlier studies concluding that a physically active lifestyle starts to develop very early in childhood and that stability of physical activity is moderate to high along the life course from youth to adulthood [31]. It has also been shown that persistent participation in sport increases the probability of a higher level of physical activity in later life [32].

Interestingly, inattention was associated with lower odds of self-rating as being physically active in adolescence whereas the opposite was true for hyperactivity/impulsivity, even after taking possible confounders such as parent-rated physical activity at age 9/12, sex, parental education level and coexisting NDPs into account. This is in line with earlier findings in a cross-sectional study [33] and might imply that children with inattention but not hyperactivity/impulsivity are less attracted to physical activity contexts such as organized sports. Some of them are overweight and they might also have developmental coordination disorder (DCD) [15, 34]. These co-existing problems may increase difficulties to get involved in physical activity. While for some adolescents the hyperactive symptoms diminish and internalize, creating more of an inner sense of restlessness, one might speculate that for others a hyperactive personality profile at age 9/12 could persist into adolescence and even adulthood. Moreover, one might speculate that children with mainly hyperactivity at age 9/12, are less impaired in daily life concerning for example social interaction and aggression, and thus might have the capacity to ‘canalize’ or express their hyperactivity through engaging in different kinds of sports and physical activity. These children are less impaired and have more social skills to be able to cope with others in group activities.

A future line of research could be to carry out studies with our design, but in which the participants belong to clinical populations of children with ADHD. Future studies could also be directed towards exploring more specifically how physical activity can moderate the associations between symptoms of inattention and hyperactivity / impulsivity and mental health.

Based on our and others’ findings, children with more inattentive symptoms are at risk of being less physically active in adolescence compared to children with less inattentive symptoms. Since insufficient physical activity might drive inequalities in health and wellbeing, research efforts should be directed towards investigating attractive and effective ways to promote life-long physical activity for children and youths with inattention symptoms in particular. In Sweden, the financial governmental support promoting health enhancing physical activity, is primarily directed to the Swedish sports federation and its clubs, who typically do not work proactively to include children with ADHD symptoms in their activities.

In a clinical setting, it is valuable to be able to distinguish differences between patients with different symptom profiles to be able to better adjust and individualize treatments. Children with mainly inattention can be quite different from those with mainly hyperactivity/impulsivity. Our results showing that children with inattention symptoms in childhood are less likely to be physically active in adolescence, can help clinicians to be more observant concerning physical activity among those patients.

There are several unique aspects of this study. The CATSS-study is one of the most comprehensive twin studies ever performed including childhood mental and somatic health. All Swedish twins were invited to participate and the response rate was high. Moreover, this study adds to previous literature by assessing population-data on a large cohort, including NDP comorbidity, looking at sex differences, and analyzing the different subtypes of ADHD symptoms.

At age 9/12 symptoms of ADHD are most certainly best rated by parents compared to the children, and at age 15 it can be easier for the adolescent to evaluate the amount of physical activity. The questions assessing physical activity were designed by the CATSS authors and are not yet validated, although they have substantial face validity. While self-rated measures of physical activity often overestimate levels of physical activity, categorical answer modes, as used in the current study at age 15, have been shown to have superior validity as compared to more open answer modes [35]. While this self-assessment item has not been validated against device-based measures of physical activity, a similar single item showed similar correlation to devise-based physical activity (r = 0.44 and 95% CI = 0.24–0.63) as a longer questionnaire (r = 0.50 and 95% CI = 0.30–0.65) [36]. This variable was dichotomized based on the WHO recommendations, so that being active enough to fulfill the recommendations was compared to not fulfilling the recommendations. When evaluating a child’s physical and mental health at age 9, the parents are usually in a better position to judge this. Symptoms of inattention, hyperactivity/impulsivity and other neuropsychiatric problems are difficult for a child in that age to describe, and this is better assessed by adults in the child’s environment. However, at age 15 the situation is different. A teenager has more insight into his or her feelings and daily life, than the adults around. That is especially true concerning symptoms of anxiety, depression and physical activity throughout the day. Another strength of the study is that we adjusted for physical activity at age 9/12. There might still, however, be residual confounding, and future investigations might consider investigating also how childhood ADHD symptoms are related to sedentary behaviors such as screen time. However, future longitudinal investigations including device-based measures as a complement to the subjective measures of physical activity will be needed to confirm the assumption that the predictors of self-rated and device-based measures of physical activity are the same.

Our study has several limitations. First, changes over time can occur under the influence of many different factors. We have not been able to control for medication, as this type of data was not available for this cohort. Second, as this is a longitudinal study, our results only include cases where the parent (at baseline) as well as the child (at follow-up) participated. This limits the number of participants compared to a cross-sectional study. Previous research using the CATSS population has demonstrated that participants have a higher socioeconomic status compared to non-participants [16]. Hypothetically, a family with lower socioeconomic status may be less motivated to participate, which could explain some of the attrition. The results need to be interpreted with this possible bias in mind. Third, we use data from ages 9 and 12 at baseline. Twelve-year-olds are on the verge of puberty. This might possibly influence the expression of ADHD symptoms. However, age differences in ADHD level at baseline were small, why possible age effects should be small. To further investigate possible age differences, we included age as a covariate in our models. According to earlier analyses though, there are no differences between the age groups. Fourth, there was a lack of information on which parent that did the reporting, the exact age of the participants (year/month/day) and it was not possible to control for income in the analyses due to lack of this data.

Generalizability from a twin-study may be questioned, despite the fact that several studies have suggested that twins are representative of the population at large [37, 38], and also that monozygotic and dizygotic twins are similar in personality variation [39]. Using a cluster robust sandwich estimator is an often applied tool in analyses to adjust the standard errors for the nested twin data when computing regression models.

## Conclusions

The finding that children with inattention symptoms are at increased risk of being less physically active in adolescence, illustrates the importance of encouraging children and adolescents with inattention to engage in physical activity. It also highlights the importance of adapting forms of physical activity to better suit children with symptoms of inattention. Children with hyperactivity symptoms were at decreased risk of self-rating as being less physically active in adolescence. Further research beyond the scope of this study is needed to challenge these findings, also using device-based measures. Furthermore, research in clinical settings including children diagnosed with ADHD, could be of great value in designing and evaluating suitable models of supporting health enhancing physical activity for children and adolescents with ADHD.

## Data Availability

The data that support the findings of this study are available from the corresponding author upon reasonable request.

## Abbreviations

ADHD: Attention deficit hyperactivity disorder
ASD: Autism spectrum disorder
A-TAC: Autism–Tics, ADHD, and other Co-morbidities inventory
CATSS: The Child and Adolescent Twin Study in Sweden
CD: conduct disorder
DCD: Developmental coordination disorder
DSM: Diagnostic and Statistical Manual of Mental Disorders
ED: Eating disorder
LD: Learning disorder
NDPs: Neurodevelopmental problems
OCD: Obsessive compulsive disorder
ODD: Oppositional defiant disorder
TD: Tic disorder
